# Chronotropic Incompetence Limits Aerobic Exercise Capacity in Patients Taking Beta-Blockers: Real-Life Observation of Consecutive Patients

**DOI:** 10.3390/healthcare9020212

**Published:** 2021-02-16

**Authors:** Krzysztof Smarz, Maciej Tysarowski, Beata Zaborska, Ewa Pilichowska-Paszkiet, Małgorzata Sikora-Frac, Andrzej Budaj, Tomasz Jaxa-Chamiec

**Affiliations:** 1 Department of Cardiology, Centre of Postgraduate Medical Education, Grochowski Hospital, 04-073 Warsaw, Poland; mtysar@gmail.com (M.T.); zaborska@kkcmkp.pl (B.Z.); paszkiet@wp.pl (E.P.-P.); msikora-frac@wp.pl (M.S.-F.); budaj@kkcmkp.pl (A.B.); tomjch@kkcmkp.pl (T.J.-C.); 2 Department of Medicine, Rutgers University New Jersey Medical School, Newark, NJ 07103, USA

**Keywords:** adrenergic beta antagonists, chronotropic incompetence, chronotropic index, exercise capacity, exercise test, oxygen uptake

## Abstract

Background: Chronotropic incompetence in patients taking beta-blockers is associated with poor prognosis; however, its impact on exercise capacity (EC) remains unclear. Methods: We analyzed data from consecutive patients taking beta-blockers referred for cardiopulmonary exercise testing to assess EC. Chronotropic incompetence was defined as chronotropic index (CI) ≤ 62%. Results: Among 140 patients all taking beta-blockers (age 61 ± 9.7 years; 73% males), 64% with heart failure, chronotropic incompetence was present in 80.7%. EC assessed as peak oxygen uptake was lower in the group with chronotropic incompetence, 18.3 ± 5.7 vs. 24.0 ± 5.3 mL/kg/min, *p* < 0.001. EC correlated positively with CI (β = 0.14, *p* < 0.001) and male gender (β = 5.12, *p* < 0.001), and negatively with age (β = −0.17, *p* < 0.001) and presence of heart failure (β = −3.35, *p* < 0.001). Beta-blocker dose was not associated with EC. Partial correlation attributable to CI accounted for more than one-third of the variance in EC explained by the model (adjusted R^2^ = 59.8%). Conclusions: In patients taking beta-blockers, presence of chronotropic incompetence was associated with lower EC, regardless of the beta-blocker dose. CI accounted for more than one-third of EC variance explained by our model.

## 1. Introduction

Chronotropic incompetence is defined as an inadequate increase in heart rate (HR) during exercise. The presence of chronotropic incompetence is associated with poor prognosis in both symptomatic and asymptomatic patients [1,2,3,4,5,6]. The frequency of chronotropic incompetence depends on the definition used, as well as the population examined, and ranges from 9% to 89% [7]. The Euro-EX prevention trial reported that, among healthy individuals, chronotropic incompetence, assessed as an inability to achieve 80% of the age-predicted HR reserve, was present in 70% of patients [8]. While chronotropic incompetence in patients not treated with beta-blockers is correlated with reduced exercise capacity (EC) [9], data from patients taking beta-blockers are ambiguous. Furthermore, in studies aimed at assessing chronotropic response and EC, chronotropic incompetence was most commonly defined as an inability to reach 80% of CI or 80% of the maximum predicted HR regardless of beta-blockers treatment, and only a few of them were focused on patients treated with beta-blockers [10,11,12,13].

Identifying factors responsible for low EC may be relevant for patient evaluation and management [14,15,16], because of its strong predictive value for all-cause and cardiovascular mortality in patients with and without heart failure [17]. In a study of 3736 consecutive patients with normal electrocardiograms and lack of heart failure, pacemakers, or atrial fibrillation and who were taking beta-blockers (either metoprolol or atenolol), chronotropic incompetence defined as a CI ≤ 62% was an independent predictor of all-cause mortality (adjusted hazard ratio 1.94, 95% confidence interval 1.43 to 2.64, *p* < 0.0001) in a 4.5-year follow-up [3]. Based on this assumption, this cut-off value is recommended in the guidelines for chronotropic incompetence diagnosis in patients treated with beta-blockers [17]. There are scarce data regarding chronotropic incompetence and EC in patients taking beta-blockers. In a recent study by Dominguez et al. which was published in the year 2018, of 74 patients with heart failure with a preserved ejection fraction within 59 on beta-blockers, in whom chronotropic incompetence was defined as the inability to achieve 62% of the CI, the heart rate response to exercise was positively associated to the patient’s EC [18]. However, this cut-off value has not been validated in daily clinical practice in various clinical conditions.

Therefore, our study aimed to assess the relationship between chronotropic incompetence and EC in consecutive patients with various cardiac diseases treated with beta-blockers.

## 2. Methods

### 2.1. Study Population and Inclusion Criteria

We retrospectively analyzed data from consecutive patients who were referred for an exercise tolerance assessment at the Exercise Physiology Laboratory at the Cardiology Department, Centre of Postgraduate Medical Education, Grochowski Hospital, Warsaw, Poland, between January 2008 and June 2016.

**Figure 1:**
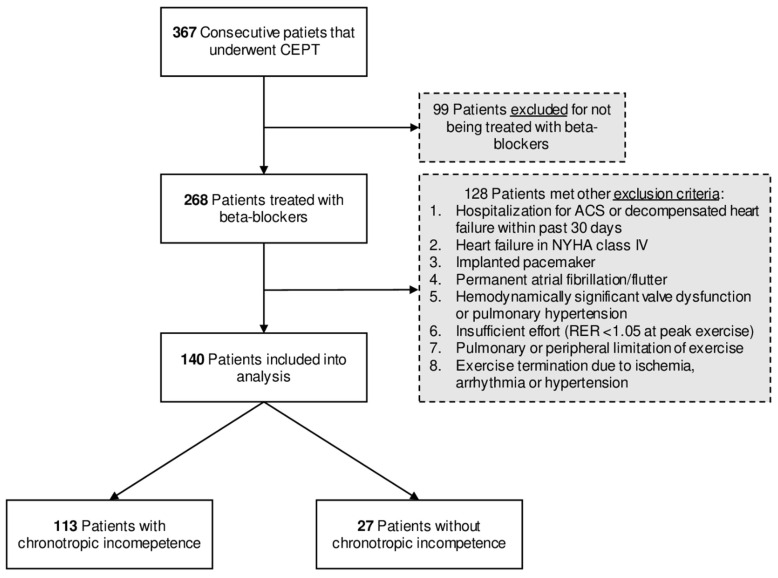
Study flow chart. Abbreviations: ACS, acute coronary syndrome; CPET, cardiopulmonary exercise test; NYHA, New York Heart Association functional classification; RER, respiratory exchange ratio.

We included patients treated with beta-blockers starting at least 4 weeks before cardiopulmonary exercise testing, as presented in Figure 1. Exclusion criteria were hospitalization for acute coronary syndromes or decompensated heart failure within the past 30 days, heart failure in the New York Heart Association functional class IV, implanted pacemakers, permanent atrial fibrillation/atrial flutter, hemodynamically significant valve dysfunction or pulmonary hypertension, insufficient effort (respiratory exchange ratio < 1.05 at peak exercise), pulmonary or peripheral limitations of exercise, stress test termination due to exercise-induced ischemia, arrhythmia, or hypertension. Patients included in the study were divided into two groups: those with and without chronotropic incompetence.

Data on patients’ demographic and clinical details, laboratory tests, medications, and comorbidities were obtained from hospital patient medical documentation. Comorbidities were established based on physicians’ diagnoses from electronic medical records. Creatinine clearance was calculated using the Cockcroft–Gault equation. Daily doses of beta-blockers were calculated as a bisoprolol-equivalent dose. Dose equivalents for beta-blockers were derived from the European Society of Cardiology Guidelines for the diagnosis and treatment of acute and chronic heart failure [19]. Doses were calculated as a bisoprolol 10 mg daily equivalent to: carvedilol 25 mg twice daily (BID); metoprolol tartrate 100 mg BID; metoprolol succinate 200 mg daily; nebivolol 10 mg daily; and sotalol 160 mg BID.

### 2.2. Echocardiography

All assessed echocardiographic studies were performed during routine evaluation by cardiologists experienced in cardiovascular imaging, and all measurements were performed according to recommendations of the American Society of Echocardiography and the European Association of Cardiovascular Imaging [20,21,22]. Patients were characterized using the following echocardiographic parameters: left ventricular end-diastolic dimension; anteroposterior dimension of the left atrium from parasternal long-axis view; basal right ventricular end-diastolic dimension and minor-axis dimension of the right atrium from four-chamber view; left ventricular ejection fraction from the biplane method of discs (modified Simpson’s rule); visual assessment of segmental contraction disturbances as (1) normal or hyperkinetic, (2) hypokinetic, (3) akinetic, and (4) dyskinetic; calculated wall motion score index (16 segments model); left ventricular diastolic dysfunction with grade 1, 2, or 3 dysfunction based on mitral inflow parameters; and right ventricular systolic dysfunction diagnosed in patients with tricuspid plane systolic excursion <17 mm.

### 2.3. Cardiopulmonary Exercise Test

All patients performed a symptom-limited cardiopulmonary exercise test using a treadmill or cycle ergometer with Schiller Cardiovit CS-200 (Schiller, Baar, Switzerland) and Ergo Spiro adapter (Garnshorn, Nederlauer, Germany), with the incremental protocol selected according to the individual’s physical condition to maintain the duration of exercise between 8 and 12 min. All patients were familiar with the exercise protocol and were encouraged to perform maximal effort (≥8 points using the 10-point Borg scale). All exercise tests were performed and analyzed by the same physician according to the guidelines of the American College of Cardiology/American Heart Association and the American Thoracic Society/American College of Chest Physicians [15,17,23,24]. The system was calibrated each day before performing the tests. Ventilation, VO_2_ uptake, and carbon dioxide output during exercise were analyzed breath by breath. VO_2_peak (mL/kg/min) was averaged from measurements taken during the last 20 s of exercise and was assessed as an EC parameter. The anaerobic threshold was calculated using a dual method approach (V-slope and ventilatory equivalent methods). Maximum predicted VO_2_ values were calculated using the Wasserman/Hansen equation [25].

Chronotropic incompetence was defined as a chronotropic index (CI) ≤ 62% [3], calculated as a percentage of a HR reserve as follows:

$$\frac{HR\;at\;peak\;exercise-Resting\;HR}{Maximum\;predicted\;HR-Resting\;HR}\times 100\%.$$

The maximum predicted HR was calculated as 220—age in years, as previously defined by Astrand et al. [26]. The percentage of predicted maximum HR achieved at peak exercise and HR reserve, defined as the change in HR from rest to peak, were also calculated.

Other analyzed cardiopulmonary exercise testing parameters included systolic blood pressure at rest and at peak exercise, ventilatory efficiency (minute ventilation versus carbon dioxide production slope), and breathing reserve at peak exercise, calculated as the percentage of maximal voluntary ventilation used [(maximal voluntary ventilation − minute ventilation at peak exercise)/maximal voluntary ventilation] × 100%. Resting spirometry parameters, calculated as forced expiratory volume in 1 s., and inspiratory vital capacity were also recorded.

### 2.4. Statistical Methods

Data were presented as mean ± standard deviation for normally distributed or median (25th and 75th percentiles) for non-normally distributed continuous variables. Categorical variables were presented as a number (percentage). Normality for all continuous variables was tested using the Shapiro–Wilk test. Group comparisons were performed using Student’s *t* test or the Mann–Whitney test for continuous variables and the χ2 (chi-squared) test for categorical variables. Univariate and multivariate linear regression analyses were performed to establish the association between independent variables and EC. The VO_2_peak (mL/kg/min) was used as the dependent variable for all models. Logarithmic transformation was used for non-normally distributed variables when analyzed in regression models. Variables for the univariate and multivariate models were selected using the Akaike Information Criterion and stepwise linear regression model. Variables with well-known effects on EC were forced into the model. All statistical tests were two-sided. Statistical significance was established as α = 0.05, and all statistical analyses were performed using R Statistical Software version 3.6.1.

## 3. Ethics

This study was conducted following the requirements set out in the Declaration of Helsinki. All patients provided written consent to take part in the cardiopulmonary exercise test. The study protocol was approved by the Centre of Postgraduate Medical Education’s Institutional Review Board, and individual consent to participate in retrospective anonymous data analysis was waived.

## 4. Results

Among 367 evaluated patients, 268 were treated with beta-blockers. The screening process was as follows: initially, from medical records, we excluded patients with submaximal exercise tests (acute coronary syndromes or decompensation of heart failure within the preceding 30 days, patients in the New York Heart Association functional class IV *n* = 37), patients with implanted pacemakers, *n* = 27, permanent atrial fibrillation/flutter, *n* = 20, and patients with severe valvular dysfunction or pulmonary hypertension, *n* = 8. Secondly, from the stress tests results, we excluded patients with insufficient effort (RER < 1.05), pulmonary or peripheral limitations of exercise, or other symptoms leading to premature stress test termination, such as ischemia, arrhythmia, or abnormal hypertensive response, *n* = 36. Finally, 140 patients were included into analysis.

The indication for cardiopulmonary exercise testing included EC evaluation as a part of disease severity assessment and treatment outcomes in patients with heart failure and/or as part of the qualification for cardiac rehabilitation in patients with ischemic heart disease and/or heart failure. The flow chart of patient selection is presented in Figure 1.

The baseline characteristics of the patients are presented in Table 1. Among 140 patients taking beta-blockers, the mean age was 61 ± 9.7 years, and 73% were males. There were 113 (81%) patients in the group with chronotropic incompetence, and 27 (19%) patients in the group without chronotropic incompetence. Of 89 (64%) patients with heart failure, 54 patients (39%) had preserved, 16 patients (11%) had mid-range, and 19 patients (14%) had reduced ejection fraction. Diabetes mellitus and impaired fasting glucose were more frequent in the group with chronotropic incompetence. The daily dose of beta-blockers was higher, and serum creatinine levels were lower in the group with chronotropic incompetence, and there were no differences in creatinine clearance between the two groups. No statistically significant differences were observed for other demographic and clinical parameters between the two groups. The indications for treatment with beta-blockers were heart failure, hypertension, or ischemic heart disease, with no differences between the groups.

The echocardiographic parameters are also presented in Table 1. No differences were observed for the left and right ventricular function and chamber dimensions between the two groups. The median time between echocardiography and cardiopulmonary exercise testing was 4 weeks.

Cardiopulmonary exercise testing results are shown in Table 2. The CI for all patients was 48.6% ± 17.3%, lower in the group with chronotropic incompetence 42.7% ± 13.0% vs. 73.1% ± 9.3% in the group without chronotropic incompetence. The group with chronotropic incompetence also had a significantly lower percentage of maximum predicted HR achieved at peak exercise compared with the group without chronotropic incompetence.

EC assessed as VO_2_peak was 19.4 ± 6.1 mL/kg/min for all patients, lower in the group with chronotropic incompetence compared with the group without chronotropic incompetence (18.3 ± 5.7 mL/kg/min vs. 24.0 ± 5.3 mL/kg/min, *p* < 0.001). The percentages of maximum predicted VO_2_ and VO_2_ at the anaerobic threshold were also significantly lower in the group with chronotropic incompetence. The VO_2_peak was 21.3 ± 5.3 mL/kg/min for males, and 14.3 ± 4.9 mL/kg/min for females, *p* < 0.001.

**Figure 2:**
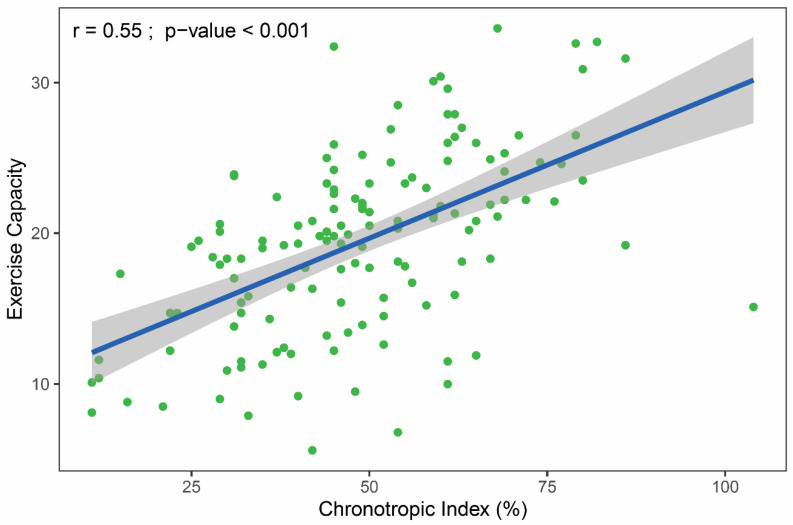
The relationship between exercise capacity (oxygen uptake at peak exercise in mL/min/kg) and chronotropic index. The gray-shaded area represents the standard error of regression line (blue). The r-value was calculated using Pearson correlation.

Figure 2 shows a moderately strong (*r* = 0.55), positive linear association between CI and EC.

The regression analysis results are presented in Table 3. Univariate regression analysis revealed that CI, male gender, treadmill exercise testing, hemoglobin concentration, and peak systolic blood pressure correlated positively with EC, while age, presence of heart failure, and diabetes mellitus/impaired fasting glucose were negatively correlated. Multivariate analysis revealed positive correlations with EC which remained for the CI and male gender, and negative correlations for the age and presence of heart failure. The beta-blocker dose was not independently associated with EC. The partial correlation attributable to CI (partial R^2^ = 24.7%) accounted for more than one-third of the variance in EC explained by the model (model adjusted R^2^ = 59.8%).

## 5. Discussion

The relationship between beta-blockers, chronotropic response, and EC is currently under investigation, and there are many ambiguities regarding this issue [27].

In our study of consecutive patients taking beta-blockers with a wide spectrum of diseases (including heart failure (64%) and chronic coronary disease (82%)) who were referred for cardiopulmonary exercise testing to assess EC, CI was common (81%) and was related to lower EC.

This study revealed that in patients taking beta-blockers, CI was the strongest independent predictor of EC, and accounted for one-third of the EC variability explained by the multivariable model. Other factors, such as gender, age, and presence of heart failure were also independent predictors of EC, although to a lesser extent. Our model revealed that echocardiographic parameters, hemoglobin and creatinine levels, the modality of the exercise test, and beta-blocker dose had no discernible effects on EC among our study population. Age, gender, body mass index, physical activity, smoking, and many comorbidities were previously examined as independently related to EC [28]. Our results confirmed previous findings that CI correlated linearly with VO_2_peak (Figure 2) [5,29].

To the best of our knowledge, our study is the first investigation on which factors and to what extent we can predict EC in non-preselected patients taking beta-blockers.

Our findings also revealed significant differences in EC with regard to chronotropic incompetence defined as a CI cut-off value of 62%. This cut-off value has previously been shown to have prognostic significance in patients treated with beta-blockers [3].

The relationship between chronotropic incompetence and EC was also investigated by Magri et al. in 549 congestive heart failure patients taking beta-blockers. The authors concluded that chronotropic incompetence negatively correlates with EC regardless of beta-blocker daily dose. However, in this study, chronotropic incompetence was diagnosed as an inability to achieve 80% of CI or 80% of maximum predicted HR [11].

Our results do not reveal a correlation between beta-blocker dose and EC, although it had been previously shown that high doses of beta-blockers can cause, aggravate, or reveal latent chronotropic incompetence [7,30,31].

Although some studies presented that treatment with beta-blockers could improve the chronotropic response by decreasing sympathetic tone and/or by increasing beta-receptor activity [32], improperly adjusted beta-blocker doses could worsen exercise capacity by decreasing heart rate response to increasing workload [33].

Our goal was to assess the relationship between chronotropic incompetence and EC in patients on beta-blockers with sinus rhythm that achieved a peak of RER ≥ 1.05, as it was considered for a lower range for valuable peak VO_2_ assessment [34]. Because of symptoms or insufficient effort, patients with premature exercise test termination were not included. Including consecutive patients with maximal effort and various diseases is a particular strength of our study. Attenuated exercise cardiac output, which is responsible for cardiac mechanisms of exercise intolerance, can be caused by an impaired chronotropic response. Treatment with beta-blockers could reveal this mechanism.

We confirmed a hypothesis that impaired exercise tolerance is related to chronotropic insufficiency in patients with various diseases treated with beta-blockers in real-life observation. Using a prognostic cut-off value of the CI could be useful for the diagnosis of chronotropic incompetence, and can explain some degree of exercise intolerance in patients on beta-blockers.

Our study has several limitations, as this was a single-center, observational, retrospective study with a relatively small group of mostly male patients. It included a diverse group of patients with and without heart failure. Brain natriuretic peptide plasma concentration values were not available in all patients, and therefore were not included in the analyses. We used an Astrand formula as it is routinely used in our hospital to calculate maximum predicted HR and calculate CI. Using other formulas could cause different results. Among the analyzed patients, some exercise tests were performed on a treadmill, some on a cycle ergometer, and different exercise protocols were used, which could have had an impact on the chronotropic response and VO_2_peak. Exercise tests on the treadmill can cause a higher peak VO_2_ uptake than on the cycle ergometer. We corrected for differences in treadmill versus a cycle ergometer test in multivariable analysis. A univariate analysis exercise on a treadmill versus a cycle ergometer correlated positively with peak VO_2_ uptake, but in multivariable analysis it did not.

Because of patients’ comorbidities and treatment with drugs other than beta-blockers, influence on chronotropic response and EC of concomitant medications could not be excluded. Use of heart rate lowering drugs other than beta-blockers in the analyzed group was low (only 6%) without differences between groups with and without chronotropic incompetence. Concomitant medication did not significantly differ between groups.

It was also shown previously that endurance training during cardiac rehabilitation can improve chronotropic response in patients taking beta-blockers [13]. Therefore, daily physical activity status can contribute to the chronotropic response and EC. In our study, data on physical activity status and quality of life were not available for all patients, and were therefore not included in our analyses.

## 6. Conclusions

In consecutive non-selected patients taking beta-blockers referred for cardiopulmonary exercise testing, chronotropic incompetence, calculated as CI, was found to be an independent predictor of reduced EC. CI positively and independently correlated with EC with the highest explained variance. Therefore, we recommend that for patients taking beta-blockers, CI is incorporated as an exercise testing parameter also in regular exercise stress tests. Our study revealed that beta-blocker dose was not an independent predictor of EC.

Correlations between chronotropic response to exercise and beta-blocker daily doses need to be evaluated in further prospective studies. We are planning a long-term follow-up of this study group to evaluate predictors of mortality.

## Figures and Tables

**Table 1:** Baseline characteristics of study participants.

	All Patients (*n* = 140)	Chronotropic Incompetence ^a^		*p*-Value
		Yes (*n* = 113)	No (*n* = 27)	
Demographics				
Age, years	61.0 ± 9.7	60.7 ± 10.0	62.1 ± 8.5	0.525
Male sex, *n* (%)	102 (73)	78 (69)	24 (89)	0.065
BMI, kg/m^2^	27.8 ± 4.1	27.8 ± 4.0	27.7 ± 4.5	0.953
Comorbidity, *n* (%)				
Chronic heart failure	89 (64)	74 (65)	15 (56)	0.514
NYHA functional class				
I	71 (51)	59 (52)	12 (44)	0.609
II	8 (6)	7 (6)	1 (4)	0.968
III	10 (7)	8 (7)	2 (7)	0.968
CAD	115 (82)	94 (83)	21 (78)	0.704
MI	110 (79)	90 (80)	20 (74)	0.709
Coronary angiography	118 (84)	96 (85)	22 (81)	0.880
PCI	107 (76)	87 (77)	20 (74)	0.945
CABG	5 (4)	4 (4)	1 (4)	1
DM/IFG	29 (20)	28 (24)	1 (4)	0.033
Hypertension	88 (63)	74 (65)	14 (52)	0.273
Current smoker	42 (30)	35 (31)	7 (26)	0.779
Paroxysmal atrial fibrillation	14 (10)	12 (11)	2 (7)	0.886
Biochemistry				
Hemoglobin, g/L	13.8 ± 1.5	13.8 ± 1.5	13.8 ± 1.5	0.934
Serum creatinine (IQR), mg/dL	0.9 (0.8–1.1)	0.9 (0.8–1.1)	1.1 (0.9–1.3)	0.005
Creatinine clearance, mL/min/1.73 m^2^	95 ± 30	97 ± 31	88 ± 29	0.188
Medication, *n* (%)				
Bisoprolol	77 (55)	67 (59)	10 (37)	0.061
Metoprolol	49 (35)	35 (31)	14 (52)	0.069
Carvedilol	9 (6)	7 (6)	2 (7)	1
Sotalol	2 (1)	2 (2)	0 (0)	NA
Nebivolol	2 (1)	1 (1)	1 (4)	0.837
Other heart rate lowering drugs ^b^	8 (6)	6 (5)	2 (7)	0.950
Dihydropyridine CCB	18 (13)	16 (14)	2 (7)	0.554
ACEI/ARB	111 (79)	92 (81)	19 (70)	0.313
Diuretics	35 (25)	30 (27)	5 (19)	0.536
BB dose, bisoprolol equivalent (IQR), mg	2.5 (2.5–5)	2.5 (2.5–5.0)	2.5 (1.2–2.5)	0.033
Echocardiography				
LV end-diastolic dimension, mm	47 (44–50)	46.0 (44–50)	47 (44–50)	0.523
Left atrium dimension, mm	40 ± 5	40 ± 5	39 ± 5	0.558
LVEF, %	53.3 ± 11.6	53.4 ± 11.9	52.8 ± 10.5	0.816
WMSI (IQR)	1.4 (1.1–1.7)	1.4 (1.1–1.8)	1.5 (1.0–1.6)	0.940
LV diastolic dysfunction, *n* (%)				0.702
Grade 1	114 (81)	90 (80)	24 (89)	0.404
Grade 2	8 (6)	7 (6)	1 (4)	0.968
Grade 3	2 (1)	2 (2)	0 (0)	NA
MR moderate, *n* (%)	33 (24)	25 (22)	8 (30)	0.567
RV end-diastolic dimension (IQR), mm	34 (30–36)	34 (29–36)	35 (32–38)	0.096
Right atrium dimension, mm	35 ± 6	36 ± 6	34 ± 5	0.267
RV systolic dysfunction, *n* (%)	38 (27)	33 (29)	5 (19)	0.378
TR moderate, *n* (%)	11 (8)	9 (8)	2 (7)	1

Note: Values represent mean ± SD, median (IQR; 25th–75th percentiles), or number (%). Bold values indicate statistical significance. Abbreviations: ACEI/ARB, angiotensin-converting enzyme inhibitor/angiotensin receptor blocker; BB, beta-blocker; BMI, body mass index; CABG, coronary artery bypass grafting; CAD, coronary artery disease; CCB, calcium channel blocker; DM/IFG, diabetes mellitus/impaired fasting glucose; IQR, interquartile range; LV, left ventricle; LVEF, left ventricular ejection fraction; MI, history of myocardial infarction; MR, mitral regurgitation; NYHA, New York Heart Association functional classification; PCI, percutaneous coronary intervention; RV, right ventricle; TR, tricuspid regurgitation; WMSI, wall motion score index. ^a^ defined as chronotropic index ≤ 62%. ^b^ non-dihydropyridine calcium channel blockers, amiodarone, propafenone, ivabradine, and digoxine.

**Table 2:** Cardiopulmonary exercise testing parameters of study participants.

	All Patients (*n* = 140)	Chronotropic Incompetence ^a^		*p*-Value
		Yes (*n* = 113)	No (*n* = 27)	
Treadmill exercise test, *n* (%)	109 (78)	86 (76)	23 (85)	0.446
Cycle ergometer exercise test, *n* (%)	31 (22)	27 (24)	4 (15)	0.446
VO_2_ at anaerobic threshold, mL/kg/min	13.7 ± 3.7	13.2 ± 3.7	16.0 ± 2.9	<0.001
VO_2_ at peak, mL/kg/min	19.4 ± 6.1	18.3 ± 5.7	24.0 ± 5.3	<0.001
VO_2_ at peak, mL/kg/min % predicted	73 ± 19	69 ± 17	89 ± 18	<0.001
CO_2_ at peak, L/min	1.8 ± 0.8	1.6 ± 0.7	2.4 ± 0.9	<0.001
METs at peak	5.5 ± 1.7	5.2 ± 1.6	6.9 ± 1.5	<0.001
HR at rest (IQR), bpm	72 (64–83)	72 (64–82)	77 (67–86)	0.091
HR at anaerobic threshold, bpm	97 ± 13	95 ± 13	106 ± 10	<0.001
HR at peak, bpm	115 ± 17	110 ± 15	136 ± 10	<0.001
HR at peak, % predicted	72 ± 10	69 ± 8	86 ± 4	<0.001
Chronotropic index, %	48.6 ± 17.3	42.7 ± 13.0	73.1 ± 9.3	<0.001
SBP at rest, mmHg	127 ± 13	127 ± 13	130 ± 13	0.289
SBP at peak (IQR), mmHg	170 (155–180)	170 (150–180)	180 (160–190)	0.075
RER at peak (IQR)	1.10 (1.05–1.16)	1.09 (1.05–1.16)	1.14 (1.06–1.21)	0.072
Min. ventilation vs. CO_2_ slope (IQR)	24 (22–28)	25 (23–28)	23.6 (21–25)	0.095
Breathing reserve at peak (IQR), %	45 (25–57)	46 (44–50)	47 (44–50)	0.523
FEV 1/IVC, % predicted	93 ± 21	93 ± 20	94 ± 23	0.841

Note: Values represent mean ± SD, median (IQR; 25th–75th percentiles), or number (%). Bold values indicate statistical significance. Abbreviations: FEV 1/IVC, forced expiratory volume in the first second/inspiratory vital capacity; HR, heart rate; IQR, interquartile range; METs, metabolic equivalents; RER, respiratory exchange ratio; SBP, systolic blood pressure; VCO_2_, carbon dioxide production; VO_2_, oxygen uptake. ^a^ defined as chronotropic index ≤ 62%.

**Table 3:** Results of univariate and multivariate linear regression analyses assessing predictors of exercise capacity (EC) measured as oxygen uptake at peak exercise (VO_2_peak, mL/min/kg).

	Univariate Analysis			Multivariate Analysis			
	β Regression Coefficient ^a^	95% CI	*p*-Value	β regression Coefficient ^a^	95% CI	*p*-Value	Explained Variance (%) ^b^
Chronotropic index, %	0.20	0.15 to 0.24	<0.001	0.14	0.09 to 0.18	<0.001	24.7
Male gender	7.01	5.05 to 8.97	<0.001	5.12	2.86 to 7.38	<0.001	14.0
Age, years	−0.22	−0.32 to −0.13	<0.001	−0.17	−0.26 to −0.09	<0.001	12.9
Heart failure	−4.55	−6.52 to −2.60	<0.001	−3.35	−4.97 to −1.72	<0.001	11.8
WMSI			0.483			0.050	3.1
Treadmill vs. cycle ergometer	3.92	1.56 to 6.28	0.001			0.066	2.7
BB daily dose, bisoprolol equivalent, mg			0.743			0.140	1.8
LVEF, %			0.147			0.224	1.2
Hemoglobin, g/L	0.81	0.15 to 1.48	0.017			0.298	0.9
Serum creatinine, mg/dL			0.295			0.343	0.7
LV diastolic dysfunction, grade 2 and 3			0.108			0.413	0.5
Height, cm			0.430			0.430	0.5
DM/IFG	−3.25	−5.70 to −0.80	0.010			0.560	0.3
Current smoker			0.670			0.586	0.2
RV systolic dysfunction			0.931			0.948	0.0
SBP at peak exercise, mmHg	0.05	0.00 to 0.09	0.034				
Hypertension			0.137				
CAD			0.133				

Note: For multivariate analysis, R^2^ = 64.1%, adjusted R^2^ = 59.8%. Bold values indicate statistical significance. Abbreviations: BB, beta-blocker; CAD, coronary artery disease; CI, confidence interval; DM/IFG, Diabetes mellitus/impaired fasting glucose; LV, left ventricle; LVEF, left ventricular ejection fraction; RV, right ventricle; SBP, systolic blood pressure; WMSI, wall motion score index. ^a^ non-standardized. ^b^ calculated as partial R^2^.

## Data Availability

The complete raw dataset file was generated on 26 January 2020 and can be accessed via the Mendeley Data repository: https://data.mendeley.com/datasets/w2jf6fr8hg/draft?a=4abca7bb-b565-4b03-bbee-f5598f56282d (accessed on 16 February 2021).

## Abbreviations

CI	chronotropic index
EC	exercise capacity
HR	heart rate
VO_2_	oxygen uptake

## References

[1] Lauer M.S., Francis G.S., Okin P.M., Pashkow F.J., Snader C.E., Marwick T.H. (1999). Impaired Chronotropic Response to Exercise Stress Testing as a Predictor of Mortality. JAMA.

[2] Azarbal B., Hayes S.W., Lewin H.C., Hachamovitch R., Cohen I., Berman D.S. (2004). The incremental prognostic value of percentage of heart rate reserve achieved over myocardial perfusion single-photon emission computed tomography in the prediction of cardiac death and all-cause mortality. J. Am. Coll. Cardiol..

[3] Khan M.N., Pothier C.E., Lauer M.S. (2005). Chronotropic Incompetence as a Predictor of Death Among Patients with Normal Electrograms Taking Beta Blockers (Metoprolol or Atenolol). Am. J. Cardiol..

[4] Myers J., Tan S.Y., Abella J., Aleti V., Froelicher V.F. (2007). Comparison of the chronotropic response to exercise and heart rate recovery in predicting cardiovascular mortality. Eur. J. Cardiovasc. Prev. Rehabil..

[5] Dobre D., Zannad F., Keteyian S.J., Stevens S.R., Rossignol P., Kitzman D.W., Landzberg J., Howlett J., Kraus W.E., Ellis S.J. (2013). Association between resting heart rate, chronotropic index, and long-term outcomes in patients with heart failure receiving β-blocker therapy: Data from the HF-ACTION trial. Eur. Heart J..

[6] Engeseth K., Hodnesdal C., Grundvold I., Liestøl K., Gjesdal K., Kjeldsen S.E., Erikssen J.E., Bodegard J., Skretteberg P.T. (2016). Temporal Reduction in Chronotropic Index Predicts Risk of Cardiovascular Death Among Healthy Middle-Aged Men: A 28-Year Follow-Up Study. J. Am. Heart Assoc..

[7] Brubaker P.H., Kitzman D.W. (2011). Chronotropic Incompetence. Circulation.

[8] Laforgia P., Bandera F., Alfonzetti E., Guazzi M. (2020). Exercise chronotropic incompetence phenotypes the level of cardiovascular risk and exercise gas exchange impairment in the general population. An analysis of the Euro-EX prevention trial. Eur. J. Prev. Cardiol..

[9] Vallebona A., Gigli G., Orlandi S., Reggiardo G. (2005). Heart rate response to graded exercise correlates with aerobic and ventilatory capacity in patients with heart failure. Clin. Cardiol..

[10] Jorde U.P., Vittorio T.J., Kasper M.E., Arezzi E., Colombo P.C., Goldsmith R.L., Ahuja K., Tseng C.-H., Haas F., Hirsh D.S. (2008). Chronotropic incompetence, beta-blockers, and functional capacity in advanced congestive heart failure: Time to pace?. Eur. J. Heart Fail..

[11] Magrí D., Palermo P., Cauti F.M., Contini M., Farina S., Cattadori G., Apostolo A., Salvioni E., Magini A., Vignati C. (2010). Chronotropic Incompentence and Functional Capacity in Chronic Heart Failure: No Role of β-Blockers and β-Blocker Dose. Cardiovasc. Ther..

[12] Al-Najjar Y., Witte K.K., Clark A.L. (2012). Chronotropic incompetence and survival in chronic heart failure. Int. J. Cardiol..

[13] Takano N., Takano H., Fukuda T., Kikuchi H., Oguri G., Fukumura K., Iwasawa K., Nakajima T. (2015). Relationship between chronotropic incompetence and β-blockers based on changes in chronotropic response during cardiopulmonary exercise testing. IJC Heart Vasc..

[14] Arena R., Myers J., Williams M.A., Gulati M., Kligfield P., Balady G.J., Collins E., Fletcher G. (2007). Assessment of Functional Capacity in Clinical and Research Settings. Circulation.

[15] Guazzi M., Adams V., Conraads V.M., Halle M., Mezzani A., Vanhees L., Arena R., Fletcher G.F., Forman D.E., Kitzman D.W. (2012). Clinical Recommendations for Cardiopulmonary Exercise Testing Data Assessment in Specific Patient Populations. Circulation.

[16] Jelinek M., Hossack K. (2019). Implications of Cardio-Respiratory Fitness on the Performance of Exercise Tests. Heart Lung Circ..

[17] Fletcher G.F., Ades P.A., Kligfield P., Arena R., Balady G.J., Bittner V.A., Coke L.A., Fleg J.L., Forman D.E., Gerber T.C. (2013). Exercise Standards for Testing and Training. Circulation.

[18] Domínguez E., Palau P., Núñez E., Ramón J.M., López L., Melero J., Bellver A., Santas E., Chorro F.J., Núñez J. (2018). Heart rate response and functional capacity in patients with chronic heart failure with preserved ejection fraction. ESC Heart Fail..

[19] Ponikowski P., Voors A.A., Anker S.D., Bueno H., Cleland J.G.F., Coats A.J.S., Falk V., González-Juanatey J.R., Harjola V.-P., Jankowska E.A. (2016). 2016 ESC Guidelines for the diagnosis and treatment of acute and chronic heart failure: The Task Force for the diagnosis and treatment of acute and chronic heart failure of the European Society of Cardiology (ESC)Developed with the special contribution of the Heart Failure Association (HFA) of the ESC. Eur. Heart J..

[20] Lang R.M., Bierig M., Devereux R.B., Flachskampf F.A., Foster E., Pellikka P.A., Picard M.H., Roman M.J., Seward J., Shanewise J.S. (2005). Recommendations for Chamber Quantification: A Report from the American Society of Echocardiography’s Guidelines and Standards Committee and the Chamber Quantification Writing Group, Developed in Conjunction with the European Association of Echocardiography, a Branch of the European Society of Cardiology. J. Am. Soc. Echocardiogr..

[21] Lang R.M., Badano L.P., Mor-Avi V., Afilalo J., Armstrong A., Ernande L., Flachskampf F.A., Foster E., Goldstein S.A., Kuznetsova T. (2015). Recommendations for Cardiac Chamber Quantification by Echocardiography in Adults: An Update from the American Society of Echocardiography and the European Association of Cardiovascular Imaging. J. Am. Soc. Echocardiogr..

[22] Nagueh S.F., Smiseth O.A., Appleton C.P., Byrd B.F., Dokainish H., Edvardsen T., Flachskampf F.A., Gillebert T.C., Klein A.L., Lancellotti P. (2016). Recommendations for the Evaluation of Left Ventricular Diastolic Function by Echocardiography: An Update from the American Society of Echocardiography and the European Association of Cardiovascular Imaging. J. Am. Soc. Echocardiogr..

[23] Committee Members Gibbons R.J., Balady G.J., Bricker J.T., Chaitman B.R., Fletcher G.F., Froelicher V.F., Mark D.B., McCallister B.D., Mooss A.N. (2002). ACC/AHA 2002 Guideline Update for Exercise Testing: Summary Article. Circulation.

[24] Society A.T. (2003). American College of Chest Physicians ATS/ACCP Statement on Cardiopulmonary Exercise Testing. Am. J. Respir. Crit. Care Med..

[25] Wasserman K., Hansen J.E., Sue D.Y., Stringer W.W., Sietsema K.E., Sun X., Whipp B.J. (2012). Normal Values. Principles of Exercise Testing and Interpretation Including Pathophysiology and Clinical Applications.

[26] Astrand I. (1960). Aerobic work capacity in men and women with special reference to age. Acta Physiol. Scand. Suppl..

[27] Palau P., Seller J., Dominguez E., Gomez I., Ramon J.M., Sastre C., de la Espriella R., Santas E., Minana G., Chorro F.J. (2020). Beta-blockers withdrawal in patients with heart failure with preserved ejection fraction and chronotropic incompetence: Effect on functional capacity rationale and study design of a prospective, randomized, controlled trial (The Preserve-HR trial). Clin. Cardiol..

[28] Alotaibi J.F., Doherty P. (2017). Evaluation of determinants of walking fitness in patients attending cardiac rehabilitation. BMJ Open Sport Exerc. Med..

[29] Jamil H.A., Gierula J., Paton M.F., Byrom R., Lowry J.E., Cubbon R.M., Cairns D.A., Kearney M.T., Witte K.K. (2016). Chronotropic Incompetence Does Not Limit Exercise Capacity in Chronic Heart Failure. J. Am. Coll. Cardiol..

[30] Gauri A.J., Raxwal V.K., Roux L., Fearon W.F., Froelicher V.F. (2001). Effects of chronotropic incompetence and β-blocker use on the exercise treadmill test in men. Am. Heart J..

[31] Witte K.K., Cleland J.G.F., Clark A.L. (2005). Chronic heart failure, chronotropic incompetence, and the effects of blockade. Heart.

[32] Vittorio T.J., Lanier G., Zolty R., Sarswat N., Tseng C.-H., Colombo P.C., Jorde U.P. (2010). Association between Endothelial Function and Chronotropic Incompetence in Subjects with Chronic Heart Failure Receiving Optimal Medical Therapy. Echocardiography.

[33] De Pauw M., Van Heuverswyn F., Duytschaever M., De Buyzere M. (2013). Chronotropic incompetence: Real life observations of a theoretical concept. Acta Cardiol..

[34] Mehra M.R., Canter C.E., Hannan M.M., Semigran M.J., Uber P.A., Baran D.A., Danziger-Isakov L., Kirklin J.K., Kirk R., Kushwaha S.S. (2016). The 2016 International Society for Heart Lung Transplantation listing criteria for heart transplantation: A 10-year update. J. Heart Lung Transplant..

